# Incidence and Prognostic Significance of Hormonal Receptors and HER2 Status Conversion in Recurrent Breast Cancer: A Retrospective Study in a Single Institute

**DOI:** 10.3390/medicina61040563

**Published:** 2025-03-22

**Authors:** Einas M. Yousef, Abdullah Mansour Alswilem, Zahrah S. Alfaraj, Danya J. Alhamood, Ghfran K. Ghashi, Hanan S. Alruwaily, Shouq S. Al Yahya, Eyad Alsaeed

**Affiliations:** 1 College of Medicine, Alfaisal University, Riyadh 11533, Saudi Arabia; 2 Oncology Center, King Saud University Medical City, Riyadh 19910, Saudi Arabia; aalswailem1@ksu.edu.sa; 3 College of Medicine, Dar Aluloom University, Riyadh 13314, Saudi Arabia; 22111564@du.edu.sa (Z.S.A.); 22111331@du.edu.sa (D.J.A.); 22111881@du.edu.sa (G.K.G.); 22111049@du.edu.sa (H.S.A.); 2218998@du.edu.sa (S.S.A.Y.); 4 Department of Radiation Oncology, College of Medicine, King Saud University, Riyadh 11411, Saudi Arabia; ealsaeed@ksu.edu.sa

**Keywords:** breast cancer, receptor conversion, HER2 status, prognostic significance, survival analysis, estrogen receptor, progesterone receptor

## Abstract

*Background and Objectives*: Changes in biomarker status are not rare and usually occur in an unfavorable direction. Evaluating changes in biomarker status is advantageous for assessing treatment options and prognosticating patients. Currently, only a few studies have explored the association between biomarker conversion and breast cancer relapse. In this study, we sought to determine the incidence of receptor conversions in patients diagnosed with recurrent breast cancer in comparison to their corresponding primary tumors and to evaluate possible influencing factors. Moreover, we aimed to assess the prognostic significance of biomarker conversion, if any was detected, in breast cancer patients. *Materials and Methods*: A retrospective cohort study was conducted among breast cancer patients treated at King Khalid University Hospital, Riyadh, Saudi Arabia. Data were collected from recurrent breast cancer patients about different parameters to assess the incidence of hormonal receptors and human epidermal growth factor 2 (HER2) status conversion between primary and recurrent tumors. The calculation of progression-free survival (PFS)/ relapse-free survival (RFS) and the overall survival (OS) was conducted to assess the prognostic value of the assessed biomarker conversion. *Results*: Progesterone receptor (PR) conversion had the highest incidence (29.9%), followed by HER2 (23%) and estrogen receptor (ER) (12.6%). Menopausal status and concurrent receptor conversion were significant factors influencing receptor status changes. However, no significant associations were found between receptor conversion and other clinical factors, including tumor stage and histological subtype. The survival analysis revealed no statistically significant differences in OS or RFS between patients with and without receptor conversion. *Conclusions*: Receptor conversion, particularly for PR and HER2, is common in recurrent breast cancer, emphasizing the importance of re-biopsy at recurrence to ensure accurate treatment decisions. While receptor conversion does not significantly impact survival outcomes in this cohort, further large-scale prospective studies are warranted to validate these findings and explore their clinical implications in breast cancer management.

## 1. Introduction

Breast cancer is the most common form of malignancy among women worldwide [1]. It is a highly heterogeneous disorder, encompassing various subtypes, each distinguished by its distinct morphology, behavioral pattern, and clinical implications. Treatment strategies for breast cancer are tailored according to the expression of estrogen receptor (ER), progesterone receptor (PR), human epidermal growth factor 2 (HER2), and Ki-67 (the proliferation marker) [2]. These markers are of particular importance in breast cancer, as they play a significant role in the growth and proliferation of breast cancer cells.

Receptor conversion or discordance occurs when a breast cancer tumor initially classified as hormone receptor-positive (ER+ and/or PR+ and/or HER+) loses these receptors, becoming hormone receptor-negative or vice versa [3]. The changes in biomarker status are not rare and usually occur in an unfavorable direction (e.g., ER+ to ER−). Depending on the type of conversion, oncologists may need to adjust the treatment plans, which might involve either omitting targeted therapy to minimize their potential adverse effects or initiating them to improve the clinical prognoses of those patients [4,5]. Many studies have reported receptor conversion in breast cancer patients after neoadjuvant chemotherapy [3,6]. It has been reported that the loss of hormonal receptors (ER and PR) following neoadjuvant chemotherapy was more frequent than their acquisition [7]. Additionally, receptor conversion has been shown to impact adjuvant treatment decisions, leading to adjustments such as withholding endocrine therapy in cases where tumors convert from receptor-positive to receptor-negative or initiating trastuzumab therapy when HER2-negative tumors acquire HER2 expression [3]. These findings underscore the dynamic nature of receptor expression and its implications for personalized treatment strategies in breast cancer.

Receptor conversion was also observed when comparing primary and metastatic breast cancers [8,9,10]. It has been reported that hormonal receptor conversion in metastatic breast cancer primarily exhibited positive to negative alteration, whereas Ki-67 changed predominantly from a low to high index when compared to the corresponding primary tumors [10]. However, limited research has explored receptor conversion in recurrent breast cancer. One study reported that the conversion rate was 34.8% for Ki-67, 20% for ER, 20% for PR, and 15.6% for HER2, which indicates that the characteristics of breast cancer can change over time [11]. To that extent, expanding our knowledge in this area holds promise for optimizing the therapeutic approaches and ultimately improving outcomes for patients with recurrent breast cancer.

Evaluating changes in biomarker status is advantageous not only for the aim of evaluating treatment options but also for prognosticating patients. Currently, only a few studies have explored the association between biomarker conversion and breast cancer relapse. In this study, we sought to determine the incidence of receptor conversions in patients diagnosed with recurrent breast cancer in comparison to their corresponding primary tumors and to evaluate possible influencing factors. We also aimed to assess the prognostic significance of biomarker conversion, if detected, in breast cancer patients.

## 2. Materials and Methods

### 2.1. Study Design and Data Source

This unicentric retrospective cohort study was conducted after the Institutional Review Board’s approval (IRB No. E-23-8338). Data were collected from the archives of breast cancer patients treated between January 2014 and December 2015 at King Khaled University Hospital, Riyadh, Saudi Arabia. Patient consent was waived to access the medical records in this retrospective study. Data were securely preserved, and patient confidentiality was respected throughout the investigation.

### 2.2. Patient Selection

The current study employed a non-probability convenience sampling method to enroll any female breast cancer patient who has complete archived data records and fulfilled the following inclusion criteria. Patients were eligible to be included in the current study if they were female with breast cancer, were at least 18 years old, and had a complete file and documentation (hormonal receptors and HER2 status) about the primary and local or metastatic recurrent breast cancer. Female patients were excluded if they had breast cancer without biopsied relapse, cases with inflammatory breast cancer, or if they had other types of cancers. All cases of male breast cancer were also excluded. At King Khaled University Hospital, Riyadh, Saudi Arabia, the recurrence of breast cancer patients is monitored through follow-up visits, blood work, and imaging. Routine imaging is performed every three months for the first two years and every six months during the following year. If any positive findings are found in blood work or imaging, core biopsy and immunohistochemistry are conducted for confirmation.

Before the beginning of the study, the sample size was calculated to be 100 when considering a 95% significance level, a 5% margin of error, and a prevalence of recurrent breast cancer of 7%. The calculation was performed using the Raosoft sample size calculator on 20 November 2023 [12]. However, due to the limited number of patients with biopsied relapse treated at the Oncology Department of this hospital, 1864 patient records were reviewed. Finally, only 88 patients were included in the final analysis, as they were the only ones who had a complete and well-documented file and met all the predefined inclusion criteria.

### 2.3. Data Collection Tool

Data collection for the current study was conducted using a predesigned data collection sheet, with information gathered from pathology reports of both primary and recurrent tumors for each included patient. The collected data included the following: demographic information (e.g., age and nationality), details about the patients, including age at menopause, presence of chronic disorders such as diabetes or autoimmune conditions, and family history of breast or other types of cancers. Additionally, comprehensive information about the tumors (both primary and recurrent) was collected, including the type of biopsy, tumor site, size, stage, grade, histological subtypes, lymph node involvement, vascular invasion, perineural invasion, cutaneous involvement, perinodal invasion, and metastasis (occurrence and type). Details regarding hormonal receptor status (ER, PR) and HER2 levels of the primary and relapsed tumors were also collected. Finally, data related to treatments (type, dose, duration, date of surgery, and associated complications) were included alongside information necessary for evaluating prognostic significance, such as the date of the first diagnosis, date of relapse, and, if applicable, date of death.

### 2.4. Immunohistochemistry

Immunohistochemical assessment of ER, PR, and HER2 was conducted using the formalin-fixed paraffin-embedded (FFPE) tissue blocks of primary and recurrent tumors’ needle biopsies. For evaluation of ER and PR, (anti-ER, rabbit monoclonal primary antibody; clone SP1, Ventana Medical Systems, Tucson, AZ, USA) and (anti-PR, rabbit monoclonal primary antibody; clone 1E2; Ventana Medical Systems, Tucson, AZ, USA) were used, respectively. To assess HER2 and Ki-67, (anti-HER2/neu, rabbit monoclonal primary antibody; clone 4B5; Ventana Medical Systems, Tucson, AZ, USA) and (RMA-0542, SP6, Lab Vision Corporation, Fremont, CA, USA) were used. All immunohistochemical staining was performed according to the manufacturer’s recommendations of the automated Benchmark XT platform (Ventana Medical Systems).

ER, PR, and HER2 immunohistochemical staining were scored using the American Society of Clinical Oncology (ASCO) and the College of American Pathologists (CAP) guidelines [13,14]. Estrogen and progesterone receptor staining were considered positive if positive nuclear staining of ≥1% of invasive tumor cells was detected. For HER2 scoring, membranous staining of invasive tumor cells was assessed, with scores ranging from 0 to 3+ according to CAP recommendation. Scores of 0 and 1+ were considered HER2-negative; a score of 3+ was considered HER2-positive, and a score of 2+ was considered equivocal. All cases with a score of 2+ were further evaluated using the fluorescence in situ hybridization (FISH) method. Scoring of Ki-67 was conducted by counting the positively stained nuclei of invasive tumor cells. A Ki-67 score ≥ 14% indicates high expression, whereas a Ki-67 score < 14% indicates low expression, following the recommendations of the Breast Cancer Working Group and St. Gallen group (2011) accepted by this Pathology Department [15].

### 2.5. Statistical Analysis

Descriptive statistics were used to summarize the demographic and clinical characteristics of the study population. Continuous variables, such as age at primary diagnosis and recurrence, were presented as means with standard deviations or medians with ranges, depending on data distribution. Categorical variables were expressed as frequencies and percentages, including tumor size, stage, histological subtypes, and receptor conversion rates. For inferential analysis, chi-square tests were employed to assess associations between categorical variables, such as receptor conversion status (ER, PR, and HER2), and factors like menopausal status, tumor stage, and treatment types. Binary logistic regression was performed to identify predictors of receptor conversions, with odds ratios (OR) and 95% confidence intervals (CI) reported for significant associations.

Survival analyses, including Kaplan–Meier (KM) survival curves and log-rank (Mantel–Cox) tests, were used to evaluate the impact of receptor conversions on time-to-death outcomes. Overall survival (OS) was defined as the time from the date of diagnosis to the date of death from any cause or the last follow-up. Relapse-free survival (RFS) was defined as the time from the completion of primary treatment to the date of tumor recurrence, metastasis, or last follow-up in the absence of recurrence. All statistical analyses were performed using the IBM SPSS Statistics 21.0 statistics program, with a significance level set at *p* < 0.05.

## 3. Results

### 3.1. Demographic and Clinical Characteristics of Study Participants

Table 1 is a descriptive table summarizing the key demographic characteristics of the included participants in the current study. The mean age at primary tumor diagnosis was 47.3 years, while the mean age at recurrence was 51.95 years, with an average interval of 4.7 years between initial diagnosis and relapse. Family history of breast cancer was positive in 13.8% of patients, with 34.5% reporting a negative history, while data were missing for the rest. The tumor size data show that 47.1% of cases had an unknown tumor size, 18.4% had tumors smaller than 2 cm, 19.5% had tumors between 2 and 5 cm, and 14.9% had tumors larger than 5 cm. Regarding menopausal status, 43.7% of patients were premenopausal, 26.4% were postmenopausal, and the rest had missing information. Invasive ductal carcinoma was the most common histological subtype (67.8%) among the study participants.

Core needle biopsy was the most common diagnostic procedure, performed in 42.5% of cases, while mastectomy, which may include simultaneous diagnostic and therapeutic purposes, accounted for 20.5% of cases. Treatment approaches revealed that neoadjuvant therapy was administered to 69% of patients, indicating its widespread use in shrinking tumors before surgery. Most patients (94.3%) underwent surgical treatment, and most received various adjuvant therapeutic approaches. Specifically, 70.1% underwent adjuvant chemotherapy, 73.6% received radiotherapy for local control, 67.8% were treated with endocrinal therapy, and 44.8% received anti-HER2 therapy. These data underscore the use of different treatment approaches that are tailored to individual tumors.

### 3.2. Changing Patterns in Tumor Staging, Locoregional Progression, and Biomarker Profiles of Primary and Recurrent Breast Cancer

Table 2 presents a comparative analysis of the tumor staging, locoregional involvement, and status of ER, PR, HER2, and Ki-67 of the primary and recurrent breast tumors. At primary diagnosis, the most common stage was stage III (35.6%), followed by stage II (17.2%), with fewer cases in stage I (5.7%) and stage IV (16.1%). At recurrence, the majority of cases progressed to stage IV (73.6%), with earlier stages being rare, including stage III (6.8%) and stages I and II (2.3% each). Locoregional involvement at the initial diagnosis was predominantly localized (42.5%) or involved lymph nodes (35.6%), with distant spread to one organ (10.3%) or multiple organs (6.9%) being less common. Locoregional involvement at recurrence showed a significant shift to systemic disease, with 60.9% of cases spreading to various organs and 17.2% to one organ. In comparison, localized and lymph node involvement decreased to 10.3% and 6.9%, respectively.

Hormone receptor status showed a slight decline in positivity at recurrence, with estrogen receptor-positive tumors slightly decreased from 75.8% to 74.7% and progesterone receptor-positive tumors declining significantly from 67.8% to 50.6%. HER2 positivity increased from 32.2% in primary tumors to 36.8% in recurrent tumors, reflecting the potential evolution of tumor biology over time. The proliferation marker Ki-67 was above 14% in a small percentage of both primary (9.2%) and recurrent (8.05%) tumors, but a substantial proportion of data on Ki-67 remained unknown (42.5% for primary and 58.6% for recurrence). These findings highlight the progression of breast cancer from primary to recurrent stages, with increased aggressiveness and changes in biomarker expression. This emphasizes the need for early detection and comprehensive management strategies to avoid or delay systemic spread.

### 3.3. Incidence of ER, PR, and HER2 Receptor Conversion

**Figure 1:**
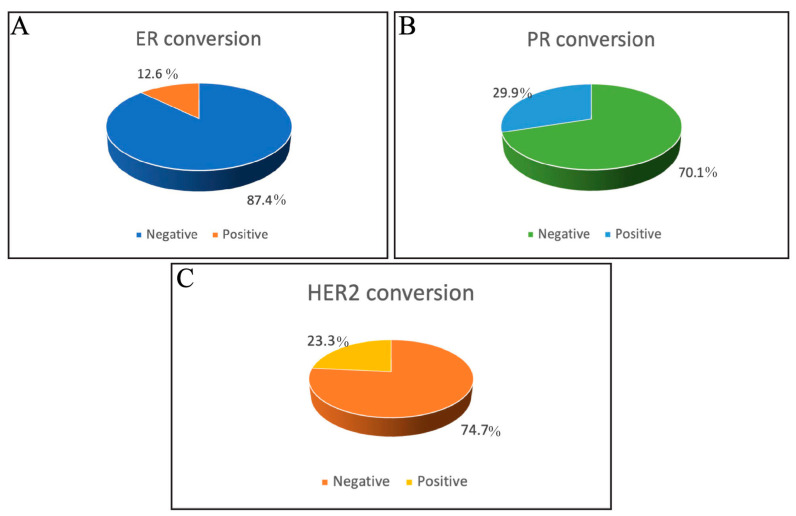
Pie charts represent the percentage of hormonal receptor and HER2 status conversion in this study’s participants. (**A**) Estrogen receptor (ER), (**B**) progesterone receptor (PR), (**C**) human epidermal growth factor receptor 2 (HER2).

Next, the incidence of hormonal receptor and HER2 status conversion was assessed among the included patients in the current study. PR conversion demonstrated the highest incidence of conversion among the three assessed receptors, as 29.9% PR conversion was observed among the assessed samples. HER2 status demonstrated a conversion rate of 23.3%, highlighting significant changes in HER2 receptor expression. On the other hand, ER conversion was the least common, occurring in only 12.6% of patients, while 87.4% of cases showed no conversion (Figure 1). This suggests that ER status remains relatively stable in most assessed cases. In addition, these results highlight the reliability of ER status for guiding hormone therapy in breast cancer management. These findings emphasize the need to re-evaluate ER, PR, and HER2 status at recurrence to identify patients who may benefit from modifying the treatment approaches, such as anti-HER2 therapies.

### 3.4. Factors Influencing the Receptor Conversion

#### 3.4.1. Univariate Analysis of Factors Affecting ER, PR, and HER2 Conversion

Various factors that might affect the conversion of ER in breast cancer patients were assessed. Among the studied variables, menopausal status demonstrated a significant association with ER conversion. Postmenopausal women had a significantly higher incidence of ER conversion compared to premenopausal women (*p* = 0.05). Among cases with positive ER conversion, 71.4% occurred in postmenopausal women, whereas 28.6% were observed in premenopausal women. PR conversion was another important factor that significantly affected the ER conversion (*p* = 0.001). Among patients with positive ER conversion, 72.7% demonstrated PR conversion, compared to 27.3% who did not experience PR conversion. Tumor stage, histological subtype, locoregional involvement of primary and recurrent tumors, BRCA1 and BRAC2 status, and HER2 do not significantly correlate with ER conversion. Hence, postmenopausal status and progesterone receptor conversion are essential factors influencing ER conversion (Table 3).

When various clinical and pathological factors that affect PR conversion were assessed, menopausal status was also found to be significantly associated with PR conversion. Our data demonstrated that postmenopausal women are more likely to have positive PR conversion (70.6%) than premenopausal cases (29.4%, *p* = 0.001). Furthermore, PR conversion was found to be significantly associated with both ER and HER2 conversions. Among patients with positive ER conversion (No. = 11), eight patients also showed PR conversion compared to only three with negative PR conversion (*p* = 0.001). Similarly, among patients with PR conversion, 56% (No. = 14) demonstrated HER2 conversion, whereas 44% (No. = 11) did not show HER2 conversion (*p* = 0.001). Postmenopausal status, HER2 conversion, and ER conversion showed significant associations with positive PR conversion. Other factors such as stage, histological subtype, the locoregional involvement of primary and recurrent tumors, and BRCA1 and BRCA2 status did not significantly affect PR conversion (Table 3).

Various factors influencing the HER2 receptor conversion in breast cancer patients were also assessed. Menopausal status was significantly associated with HER2 conversion, as postmenopausal women were more likely to experience HER2 conversion (66.7% in positive cases) compared to 33.3% in premenopausal cases (*p* = 0.04). Another essential factor that influenced the HER2 conversion was the PR conversion. Among HER2 conversion-positive cases, 68.2% also demonstrated PR conversion compared to 31.8% that did not show PR conversion. Conversely, the stage of recurrent tumor, histological subtype, locoregional involvement of primary and recurrent tumors, and BRCA1 and BRCA2 status did not significantly affect HER2 conversion.

The association between ER, PR, and HER2 conversion and different approaches to therapies was assessed. No significant association was detected between the receptor’s conversion and any of the assessed therapies, as shown in (Table 4). This indicates that none of the therapy types significantly influenced ER, PR, and HER2 conversion.

#### 3.4.2. Multivariate Analysis of Factors Affecting ER, PR, and HER2 Conversion

The binary logistic regression model was performed to identify significant predictors of hormonal receptor and HER2 status conversions to strengthen the earlier findings. Interestingly, the multivariate analysis confirmed the findings of the univariate analysis for factors affecting ER, PR, and HER2 conversion. This binary logistic regression model identifies only PR as a significant predictor of ER conversion. This analysis demonstrated that patients experiencing PR conversion were about 9.89 times more likely to have ER conversion (*p* = 0.02). Menopausal status did not significantly affect ER conversion, with premenopausal women being less likely to experience ER conversion than postmenopausal women (OR = 0.73, *p* = 0.1). While HER2 conversion shows a positive association with ER conversion (OR = 1.7), it is not statistically significant (*p* = 0.1) (Table 5).

When assessing the PR conversion, HER2 conversion showed a strong association, as patients experiencing HER2 conversion were about 15.8 times more likely to have PR conversion (*p* = 0.003). Menopausal status also significantly affects PR conversion, with premenopausal women being less likely to experience PR conversion compared to postmenopausal women (OR = 0.153, *p* = 0.019). While ER conversion positively correlates with PR conversion (OR = 7.958), it is not statistically significant (*p* = 0.076). HER2 conversion and menopausal status are key predictors of PR conversion (Table 5).

The binary logistic regression model identifies significant predictors of HER2 conversion. PR conversion is strongly associated with HER2 conversion, with patients experiencing PR conversion being about 2.52 times more likely to have HER2 conversion (*p* = 0.02). Menopausal status also significantly affects HER2 conversion, with premenopausal women less likely to experience HER2 conversion than postmenopausal women (OR = 0.11, *p* = 0.001). While ER conversion positively correlates with HER2 conversion (OR= 1.23), it is not statistically significant (*p* = 0.72). Hence, PR conversion and menopausal status are the key predictors of HER2 conversion (Table 5).

### 3.5. Prognostic Effects of Receptor Conversion

#### 3.5.1. Assessment of Overall Survival (OS)

**Figure 2:**
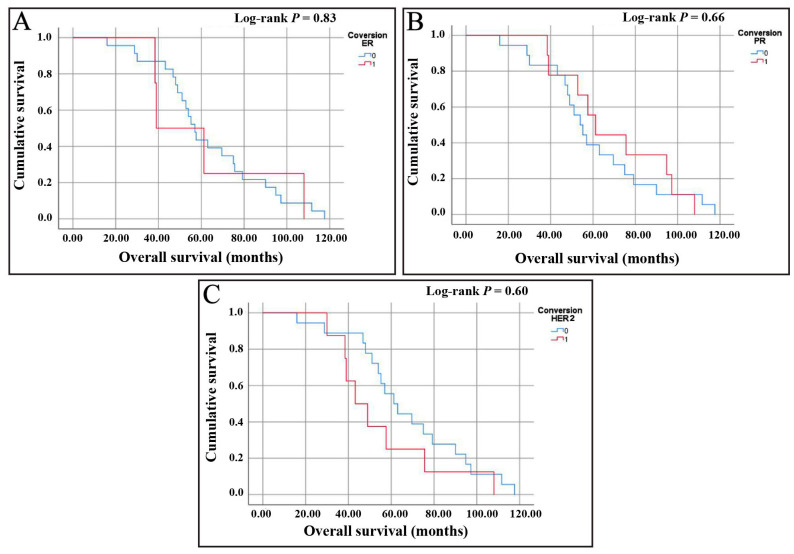
Kaplan–Meier curves showing OS based on receptor conversion status for (**A**) estrogen receptor (ER), (**B**) progesterone receptor (PR), and (**C**) human epidermal growth factor receptor 2 (HER2). The x-axis represents time, while the y-axis shows cumulative survival probability, ranging from 1 (100%) at the start of the study (when all patients are alive) to 0 (0%) when all patients have experienced death.

An OS analysis for ER conversion revealed that patients who did not experience ER conversion have a median survival time of 57 months, with a 95% CI ranging from 51.37 to 62.64 months. On the other hand, patients with ER conversion have a shorter median survival time of 39 months. However, the CI is wider, ranging from 16.66 to 61.34 months, indicating more variability in the survival data for this group. The log-rank test showed no statistically significant difference in the survival distributions between the two groups (*p*-value = 0.83). The Kaplan–Meier survival curve (Figure 2A) similarly illustrates a minimal difference between the two groups, with overlapping curves that do not suggest a clear survival advantage or disadvantage based on ER conversion status.

Next, an OS analysis of patients without PR conversion showed a median survival time of 54 months, with a 95% CI ranging from 45.27 to 62.73 months. In contrast, patients with PR conversion have a slightly higher median survival time of 61 months, with a 95% CI between 50.68 and 71.72 months. Despite the difference in median survival times, the log-rank test reveals no statistically significant difference between the survival distributions of the two groups (*p*-value = 0.66). The Kaplan–Meier survival curve (Figure 2B) also reflects that the survival curves for both groups are relatively similar, showing no apparent survival advantage for either group. The overlapping CI and the non-significant *p*-value suggest that PR conversion does not significantly impact survival time in this cohort.

Lastly, upon assessing the OS of patients without HER2 conversion, a median survival time of 61.2 months, with a 95% CI ranging from 48.73 to 73.67 months, was detected. Conversely, patients with HER2 conversion exhibit a shorter median survival time of 43.2 months, with a 95% CI extending from 29.4 to 57 months. Although there is a noticeable difference in survival times between the groups, the 95% CI overlap suggests that the distinction in median survival times is not statistically significant (*p*-value = 0.60). The Kaplan–Meier survival curve (Figure 2C) also indicates that, while there is a tendency toward reduced survival among patients with HER2 conversion, the variation within each group can account for this difference. This shows a lack of definitive statistical evidence to support a clear survival disadvantage associated with HER2 conversion.

#### 3.5.2. Assessment of Relapse-Free Survival (RFS)

**Figure 3:**
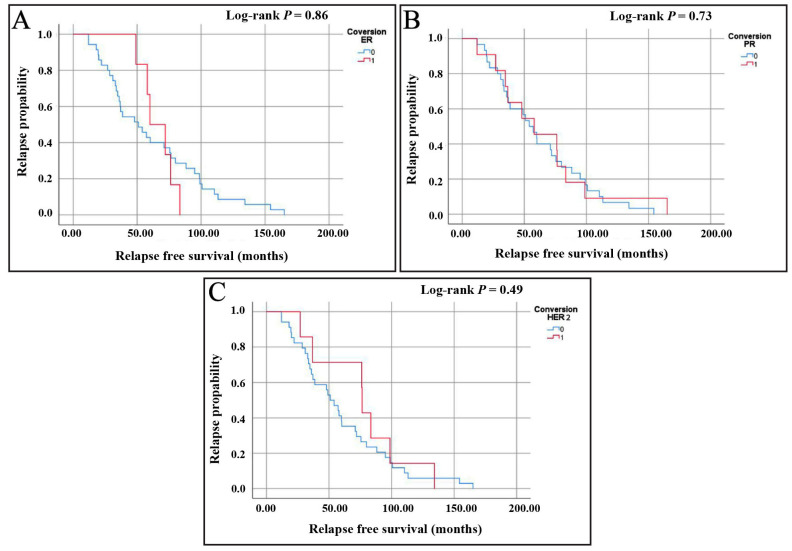
Kaplan–Meier curves showing RFS based on receptor conversion status for (**A**) estrogen receptor (ER), (**B**) progesterone receptor (PR), and (**C**) human epidermal growth factor receptor 2 (HER2).

Next, RFS has been assessed for the hormonal receptor and HER2 conversions. For ER conversion analysis, the Kaplan–Meier curve demonstrated a gradual decline in the relapse probability over time for both groups (Figure 3A). The median survival time for patients with ER conversion was 59.99 months (95% CI: 43.19–76.78), compared to 50.95 months (95% CI: 27.61–74.29) for those without conversion. Additionally, the median time to recurrence was 60 months for patients with ER conversion and 36 months for those with no ER conversion, suggesting a longer recurrence-free period for those who experienced ER conversion. Although ER conversion appears to be associated with longer RFS, the overlapping confidence intervals indicate that this difference is not statistically significant (the log-rank test, *p* = 0.86).

For PR conversion, the median survival time was 54.04 months (95% CI: 39.32–68.77) for patients without PR conversion, compared to a slightly higher median of 57.98 months (95% CI: 15.52–100.45) for those with PR conversion (Figure 3B). The median time to recurrence was 60 months for PR conversion and 36 months for no PR conversion, again suggesting a longer recurrence-free period for those who experienced PR conversion. However, the log-rank test (*p* = 0.73) indicated no statistically significant difference, suggesting that the observed findings may be due to variability rather than a true effect.

Regarding HER2 conversion, the median survival time for patients with no HER2 conversion was 50.95 months (95% CI: 37.72–64.19), compared to a higher median of 76.41 months (95% CI: 75.65–77.17) for patients with HER2 conversion (Figure 3C). Additionally, the median time to recurrence was 60 months for patients with HER2 conversion and 36 months for those without HER2 conversion. This suggested a potential tendency toward a longer recurrence-free period for patients who experienced HER2 conversion. However, the log-rank test revealed no statistically significant differences between groups (the log-rank test, *p* = 0.49), underscoring the need for cautious interpretation of these findings.

## 4. Discussion

Receptor conversion in breast cancer refers to changes in the expression of receptors such as ER, PR, and HER2 during disease progression. These receptors play crucial roles in determining the prognosis and therapeutic approaches of breast cancer. Receptor conversion has clinical significance, as it may affect response to treatment, necessitate treatment adjustments, and impact patient outcomes. So, understanding the frequency and prognostic relevance of receptor conversion is crucial for optimizing personalized treatment strategies and improving survival outcomes in breast cancer patients. This study aimed to assess the incidence, possible influencing factors, and prognostic effects of ER, PR, and HER2 conversion in recurrent breast cancer compared to their expression in the corresponding primary tumors.

The current study revealed that PR conversion showed the highest incidence (29.9%), followed by HER2 (23%) and ER (12.6%). This indicates the relative stability of ER status and reinforces its reliability in guiding hormonal therapy in breast cancer management. This low rate of ER conversion in comparison to other PR and HER2 is consistent with a previous study, which demonstrated the conversion of ER, PR, and HER2 status as 6.5%, 13.0%, and 4.4%, respectively, among 294 studied cases [16]. However, these findings contradict those of Xueyang Hu et al., who reported that out of 130 patients with metastatic breast cancer biopsies, the inconsistent expression rates of ER, PR, and HER2 in primary and metastatic lesions were 47.69%, 51.54%, and 28.10%, respectively [17]. This contradiction can be attributed to differences in study designs, patient populations, sample sizes, disease stages, methodologies for receptor evaluation, and demographics between patient cohorts.

The data presented in this study demonstrated that factors significantly associated with receptor conversion included menopausal status and another receptor conversion. In contrast, other clinical variables, such as the stage of the tumor, histological subtypes, and status of BRCA1/2, did not show any significant association. The observed association of ER, PR, and HER2 conversion in postmenopausal patients might be induced by the menopausal transition-associated hormonal changes, which may alter tumor biology. However, further investigations are needed to clarify the molecular mechanisms underlying this association. Additionally, the significant association between receptor conversion and the conversion of other receptors indicates that changes in the expression of one receptor may induce the status of another. The interconnected molecular pathways can explain this observation or crosstalk between signaling mechanisms that might induce receptor conversion [18,19]. It has been reported that both ER and PR are tightly linked and that PR is a significant determinant of ER-driven gene programs in breast cancer [20].

Additionally, HER2 signaling can modulate ER and PR activity by activating downstream pathways such as PI3K/AKT and MAPK, which can alter receptor expression or functionality [21]. Conversely, it has been shown that ER signaling can downregulate HER2 expression [22], creating a complex feedback loop. This bidirectional communication between these receptors influences tumor behavior and contributes to therapy resistance [23]. These complex interactions between these receptors reflect the dynamic nature of breast cancer and can partly explain our findings.

Our data from the prognostic analysis revealed no statistically significant differences in OS or RFS based on receptor conversion status. This contradicts the findings of previous studies, which demonstrated a significant effect of receptor conversion on OS [17,24]. This discrepancy may arise from the limited sample size in the current study. Furthermore, it can be attributable to differences in study design, patient populations, methodologies for evaluating receptor conversion, stages of the included patients, and treatment regimens among cohorts. Future research should focus on larger, multicenter cohorts to validate these findings and assess the prognostic value of receptor conversion.

The observed receptor conversions underscore the importance of re-biopsy at recurrence, as treatment decisions based on primary tumor biomarkers may no longer be valid. For instance, patients whose breast cancer converts at recurrence from being HER2 -ve to HER2 +ve may benefit from anti-HER2 therapies, even if they initially do not benefit from this approach. Similarly, ER and PR conversion rates highlight the potential need for adjustments in hormonal therapy. These findings support that breast cancer treatment has to be continually tailored to the evolving characteristics of the tumor.

This study has several limitations that need to be acknowledged. First, as a retrospective cohort study, it relied on a review of patients’ records that were not originally designed to collect data for research. So, missing data, lost follow-ups, potential selection, and recall bias may affect the results and generalizability of our findings [25]. Second, the relatively small sample size also limits the statistical power to detect subtle associations between receptor conversion and survival outcomes. Additionally, immunohistochemistry is used for receptor status assessment, which, while clinically relevant, may be influenced by interobserver variability and methodological differences in staining methods.

Future prospective research should focus on larger multicenter prospective studies to validate our findings and further explore the prognostic value of receptor conversion in recurrent breast cancer. A more in-depth exploration of the molecular mechanisms underlying receptor conversion using advanced techniques such as next-generation sequencing and proteomics may provide insight into potential therapeutic targets. Lastly, studies assessing the impact of receptor conversion on treatment resistance and long-term survival in different breast cancer subtypes would be crucial for optimizing clinical management.

## 5. Conclusions

In conclusion, this study highlights the dynamic nature of receptor expression in recurrent breast cancer, emphasizing the need for re-biopsy to guide treatment decisions. Our findings revealed that PR and HER2 conversions were more frequent than ER conversions, with menopausal status and concurrent receptor changes playing significant roles in these alterations. Additionally, no significant association was observed between receptor conversion and overall or relapse-free survival. Nonetheless, our findings suggest that reassessing recurrent breast cancer by integrating re-biopsy and biomarker analysis could help optimize individualized treatment strategies that ultimately improve patient outcomes. Although our findings provide valuable insights into receptor conversion, additional large-scale prospective studies are needed to further explore its clinical significance in relation to different subtypes of breast cancer.

## Figures and Tables

**Table 1:** Demographic and clinical data of patients included in the current study.

Variable	No (%)
Age of primary tumor *	47.3 (10.1)
Age of recurrence *	51.95 (10.6)
Difference between first diagnosis and relapse (y) *	4.7 (3.4)
Family history of breast cancer	
Positive	12 (13.8%)
Negative	30 (34.5%)
Missing	46 (51.7%)
Primary tumor size	
<2 cm	16 (18.4%)
2–5 cm	17 (19.5%)
>5 cm	13 (14.9%)
Unknown	41 (47.1%)
Menopausal status	
Postmenopausal	23 (26.4%)
Premenopausal	38 (43.7%)
Missing	26 (29.9%)
Histological subtype	
Ductal carcinoma in situ	3 (3.4%)
Invasive duct carcinoma	59 (67.8%)
Invasive adenosquamous carcinoma	2 (2.3%)
invasive lobular carcinoma	5 (5.7%)
Type of biopsy	
Core needle biopsy	37 (42.5%)
Mastectomy	18 (20.5%)
Missing	32 (36.8%)
Treatment	
Neoadjuvant	60 (69%)
Surgical	82 (94.3%)
Adjuvant chemotherapy	61 (70.1%)
Radiotherapy	64 (73.6%)
Endocrinal therapy	59 (67.8%)
Anti-HER2	39 (44.8%)

* Mean ± SD.

**Table 2:** Comparative analysis of staging, locoregional involvement, and biomarker status in primary and recurrent breast tumors.

Variable	Primary No (%)	Recurrent No (%)
Stage of the tumor		
I	5 (5.7%)	2 (2.3%)
II	15 (17.2%)	2 (2.3%)
III	31 (35.6%)	6 (6.8%)
IV	14 (16.1%)	64 (73.6%)
Missing	22 (25.3%)	12 (13.8%)
Locoregional involvement		
Localized	37 (42.5%)	9 (10.3%)
Lymph node	31 (35.6%)	6 (6.9%)
Spread to one organ	9 (10.3%)	15 (17.2%)
Spread to more than one organ	6 (6.9%)	53 (60.9%)
Missing	4 (4.6%)	4 (4.6%)
Estrogen receptor		
Positive	66 (75.8%)	65 (74.7%)
Negative	21 (24.1%)	22 (25.3%)
Progesterone receptor		
Positive	59 (67.8%)	44 (50.6%)
Negative	28 (32.2%)	43 (49.4%)
HER2		
Positive	28 (32.2%)	32 (36.8%)
Negative	56 (64.4%)	50 (57.5%)
Equivalent	3 (3.4%)	5 (5.7%)
Ki-67		
>14%	8 (9.2%)	7 (8.05%)
<14%	42 (48.3%)	29 (33.3%)
Unknown	37 (42.5%)	51 (58.6%)

**Table 3:** Univariate analysis of factors affecting ER conversion.

Variables	ER Conversion			PR Conversion			HER2 Conversion		
	Negative No (%)	Positive No. (%)	*p*-Value	Negative No (%)	Positive No. (%)	*p*-Value	Negative No (%)	Positive No. (%)	*p*-Value
**Stage of recurrent tumor**									
I	2 (3%)	0	0.71	2 (2.8%)	1 (4.3%)	0.37	1 (1.80%)	2 (11.8%)	0.15
II	1 (1.5%)	1 (10%)		2 (2.8%)	0		1 (1.80%)	2 (5.9%)	
III	6 (9.2%)	0		3 (5.7%)	3 (13%)		4 (7.20%)	2 (11.8%)	
IV	55 (84.6%)	9 (90%)		45 (86.5%)	19 (82.6%)		50 (89.3%)	12 (70.6%)	
**Menopausal status**									
Postmenopausal	18 (33.3%)	5 (71.4%)	0.05	11 (25%)	12 (70.6%)	0.001	15 (31.3%)	8 (66.7%)	0.04
Premenopausal	36 (66.7%)	2 (28.6%)		33 (75%)	5 (29.4%)		33 (68.8%)	4 (33.3%)	
**Histological subtype**									
Ductal carcinoma in situ	3 (4.8%)	0	0.89	3 (6.1%)	0	0.64	3 (5.70%)	0	0.83
Invasive duct carcinoma	63 (85.5%)	6 (85.7%)		41 (83.7%)	18 (90%)		43 (82.7%)	16 (94.1%)	
Invasive adenosquamous carcinoma	2 (3.2%)	0		2 (4.1%)	0		2 (3.80%)	0	
Invasive lobular carcinoma	4 (6.4%)	1 (14.3%)		3 (6.1%)	2 (10%)		4 (7.70%)	1 (5.90%)	
**Locoregional involvement (primary)**									
Localized	33 (45.2%)	4 (40%)	0.75	25 (43.1%)	12 (48%)	0.61	25 (41%)	10 (50%)	0.7
Lymph node	28 (38.4%)	3 (30%)		24 (41.4%)	7 (28%)		23 (37.7%)	8 (40%)	
Spread to one organ	7 (9.6%)	2 (20%)		5 (8.6%)	4(16%)		8 (13.1%)	1 (5%)	
Spread to more than one organ	5 (6.8%)	1 (10%)		4 (6.9%)	2 (8%)		5 (8.20%)	1 (5%)	
**Locoregional involvement (recurrent)**									
Localized	9 (12.50%)	0	0.24	7 (12.3%)	2 (7.7%)	0.31	5 (8.2%)	4 (20%)	0.39
Lymph node	4 (5.60%)	2 (18.2%)		4 (7%)	2 (7.7%)		4 (6.6%)	2 (10%)	
Spread to one organ	14 (19.4%)	1 (9.1%)		13 (22.8%)	2 (7.7%)		12 (19.7%)	2 (10%)	
Spread to more than one organ	45 (62.5%)	8 (72.7%)		33 (57.9%)	20 (76.9%)		40 (65.6%)	12 (60%)	
**BRCA1**									
Negative	15 (83.3%)	3 (100%)	0.45	13 (86.7%)	5 (83.3%)	0.84	15 (93.8%)	3 (60%)	0.23
Positive	3 (16.7%)	0		2 (13.3%)	1 (16.7%)		1 (6.30%)	2 (40%)	
BRCA2									
Negative	16 (88.9%)	3 (100%)	0.54	14 (93.3%)	5 (83.3%)	0.48	15 (93.8%)	4 (80%)	0.36
Positive	2 (11.15)	0		1 (6.7%)	1 (16.7%)		1 (6.3%)	1 (20%)	
**Estrogen receptor (PR) conversion**									
No	--	--		58 (95.1%)	18 (69.2%)	0.001	59 (90.8%)	17 (31.8%)	0.1
Yes	--	--		3 (4.9%)	8 (30.8%)		6 (9.2%)	5 (22.7%)	
**Progesterone receptor (PR) conversion**									
No	58 (76.3%)	3 (27.3%)	0.001	--	--		54 (83.1%)	7 (31.8%)	<0.001
Yes	18 (23.7%)	8 (72.7%)		--	--		11 (16.9%)	15 (68.2%)	
**HER2 conversion**									
No	59 (79.7%)	6 (54.5%)	0.06	54 (90%)	11 (44%)	0.001	--	--	
Yes	15 (20.3%)	5 (45.5%)		6 (10%)	14 (56%)		--	--	

**Table 4:** Association between receptor conversion and type of received therapy.

	ER Conversion			PR Conversion			HER2 Conversion		
	Negative No. (%)	Positive No. (%)	*p*-Value	Negative No. (%)	Positive No. (%)	*p*-Value	Negative No. (%)	Positive No. (%)	*p*-Value
**Neoadjuvant therapy**									
Yes	22 (28.9%)	5 (45.5%)	0.31	18 (29.5%)	9 (34.6%)	0.63	18 (27.7%)	8 (40%)	0.2
No	54 (71.10%)	6 (54.5%)		43 (70.5%)	17 (65.4%)		47 (72.3%)	12 (60%)	
**Surgical treatment**									
Yes	4 (5.30%)	1 (9.10%)	0.61	4 (6.6%)	1 (3.8%)	0.62	3 (4.6%)	2 (10%)	0.58
No	72 (94.70%)	10 (90.9%)		57 (93.4%)	25 (96.2%)		62 (95.4%)	18 (90%)	
**Chemotherapy**									
Yes	23 (30.3%)	3 (27.3%)	0.84	21 (34.4%)	5 (19.2%)	0.16	20 (30.8%)	5 (25%)	0.62
No	53 (69.7%)	8 (72.7%)		40 (65.6%)	21 (80.8%)		45 (69.2%)	15 (75%)	
**Radiotherapy**									
Yes	19 (25%)	4 (36.4%)	0.4	13 (21.3%)	10 (38.5%)	0.097	16 (24.6%)	7 (35%)	0.39
No	57 (75%)	7 (63.6%)		48 (78.7%)	16 (61.5%)		49 (75.4%)	13 (65%)	
**Hormonal therapy**									
Yes	25 (32.9%)	3 (27.3%)	0.71	22 (36.10%)	6 (23.1%)	0.23	24 (36.9%)	4 (20%)	0.16
No	51 (67.10%)	8 (72.7%)		39 (63.90%)	20 (76.9%)		41 (63.1%)	16 (80%)	

**Table 5:** Multivariate binary logistic regression for predictors of ER, PR, and HER2 conversion.

Receptor	Predictors	B (Coefficient)	*p*-Value	OR (95% CI)
ER	PR Conversion (Reference: No conversion)	2.293	0.02	9.89 (1.33–73.50)
	HER2 Conversion (Reference: No conversion)	1.703	0.1	5.49 (0.68–44.087)
	Menopausal status (Reference: Postmenopausal)	−0.309	0.15	0.734 (1.12–0.150)
PR	ER Conversion (Reference: No conversion)	2.074	0.076	7.958 (0.81–78.6)
	Menopausal status (Reference: Postmenopausal)	−1.877	0.019	0.153 (0.03–0.74)
	HER2 Conversion (Reference: No conversion)	2.763	0.003	15.84 (2.6–95.7)
HER2	ER Conversion (Reference: No conversion)	0.207	0.7	1.23 (0.39–3.84)
	PR Conversion (Reference: No conversion)	0.93	0.02	2.52 (1.12–5.69)
	Menopausal status (Reference: Postmenopausal)	−2.16	0.001	0.125 (0.76–0.17)

## Data Availability

The raw data supporting the conclusions of this article will be made available by the authors upon request.

## Abbreviations

The following abbreviations are used in this manuscript:

ASCO	American Society of Clinical Oncology
CAP	College of American Pathologists
CI	confidence intervals
ER	estrogen receptor
FFPE	formalin-fixed paraffin-embedded
FISH	fluorescence in situ hybridization
HER2	human epidermal growth factor 2
KM	Kaplan–Meier
OR	odds ratios
PR	progesterone receptor
RFS	relapse-free survival
